# Item

**Published:** 1905-03

**Authors:** 


### ITEM.

The Denver Chemical Manufacturing Company, of New York, has put out a booklet on antiphlogistine, which is a clever bit of advertising. It contains a number of reproductions in color, showing various applications of antiphlogistine for many different purposes, and is printed on heavy calendered paper, giving it an artistic appearance quite beyond the common order.

